# Cloning and expression analysis of mevalonate kinase and phosphomevalonate kinase genes associated with the MVA pathway in *Santalum album*

**DOI:** 10.1038/s41598-021-96511-4

**Published:** 2021-08-19

**Authors:** Meiyun Niu, Yuping Xiong, Haifeng Yan, Xinhua Zhang, Yuan Li, Jaime A. Teixeira da Silva, Guohua Ma

**Affiliations:** 1 Guangdong Provincial Key Laboratory of Applied Botany, South China Botanical Garden, The Chinese Academy of Sciences, Guangzhou, 510650 China; 2 University of Chinese Academy of Sciences, Beijing, 100039 China; 3 Guangxi Academy of Agricultural Sciences, Nanning, 30007 China; 4 P.O. Box 7, Miki-cho, Ikenobe 3011-2, Kagawa-ken, 761-0799 Japan

**Keywords:** Biotechnology, Molecular biology, Physiology

## Abstract

Sandalwood (*Santalum album* L.) is highly valued for its fragrant heartwood and extracted oil. Santalols, which are the main components of that oil, are terpenoids, and these are biosynthesized via the mevalonic acid (MVA) pathway. Mevalonate kinase (MK) and phosphomevalonate kinase (PMK) are key enzymes in the MVA pathway. Little is known about the genes that encode MK and PMK in *S. album* or the mechanism that regulates their expression. To isolate and identify the functional genes involved in santalol biosynthesis in *S. album*, an *MK* gene designated as *SaMK*, and a *PMK* gene designated as *SaPMK*, were cloned from *S. album*. The sequences of these genes were analyzed. A bioinformatics analysis was conducted to assess the homology of *SaMK* and *SaPMK* with *MK* and *PMK* genes from other plants. The subcellular localization of *SaMK* and *SaPMK* proteins was also investigated, as was the functional complementation of *SaMK* and *SaPMK* in yeast. Our results show that the full-length cDNA sequences of *SaMK* and *SaPMK* were 1409 bp and 1679 bp long, respectively. *SaMK* contained a 1381 bp open reading frame (ORF) encoding a polypeptide of 460 amino acids and *SaPMK* contained a 1527 bp ORF encoding a polypeptide of 508 amino acids. *SaMK* and *SaPMK* showed high homology with *MK* and *PMK* genes of other plant species. Functional complementation of *SaMK* in a MK-deficient mutant yeast strain YMR208W and *SaPMK* in a PMK-deficient mutant yeast strain YMR220W confirmed that cloned *SaMK* and *SaPMK* cDNA encode a functional MK and PMK, respectively, mediating MVA biosynthesis in yeast. An analysis of tissue expression patterns revealed that *SaMK* and *SaPMK* were constitutively expressed in all the tested tissues. *SaMK* was highly expressed in young leaves but weakly expressed in sapwood. *SaPMK* was highly expressed in roots and mature leaves, but weakly expressed in young leaves. Induction experiments with several elicitors showed that *SaMK* and *SaPMK* expression was upregulated by methyl jasmonate. These results will help to further study the role of *MK* and *PMK* genes during santalol biosynthesis in *S. album*.

## Introduction

*Santalum album* L., commonly known as Indian sandalwood, belongs to the Santalaceae and is a slow-growing, evergreen, root semi-parasitic tree widely distributed in tropical and temperate regions such as India, Sri Lanka, the Malay Archipelago, and southern China^1,2^. Sandalwood is highly valued for its fragrant heartwood and its extracted oil is used in incense, perfumes, cosmetics, pharmaceuticals, and ornamental carvings^3^. Sandalwood oil has shown a variety of biological activities, including antiviral, anticarcinogenic, antipyretic, antiseptic, antiscabietic, antitumor, and diuretic^4–6^. Sandalwood usually yields 3–7% essential oil depending on the region and hemisphere^7^. The value of a sandalwood tree depends on three important characteristics: the volume of heartwood, and the concentration and quality of its heartwood oil^8^. Specifications for sandalwood oil reported in the Food Chemicals Codex^9^ indicate that the international (ISO) standard for sandalwood oil stipulates that the minimum content of free alcohols (assessed by santalol content) should be 90%. The principal components of *S. album* essential oil distilled from heartwood are sesquiterpenoids, including four main sesquiterpene alcohols, namely α-santalol, β-santalol, *epi*-β-santalol and α-exo-bergamotol^10–12^. Previous studies showed that α-santalol and β-santalol are responsible for the pleasant fragrance of sandalwood and that α-santalol is primarily responsible for the essential oil’s bioactivity^13^. However, global sandalwood resources are diminishing due to habitat destruction and over-exploitation, and commercial-scale production has complex requirements for cultivation^7,14^, limitations that can to some extent be overcome through the use of biotechnology^15^. Therefore, biotechnological methods are necessary to improve santalol production to meet rapidly increasing commercial demands. Different strategies have been studied to increase santalol content^16^, including the treatment of *S. album* trees with exogenous substances such as 6-benzyladenine (BA), ethephon (ETH) and methyl jasmonate (MeJA)^17,18^, chemical synthesis^19–22^, heterologous expression^23–25^, in vitro culture and bioreactors^26^, and genetic transformation^27^. Metabolic engineering of the mevalonate (MVA) pathway provides an alternative approach to the traditional synthesis of terpenoids^28^.

In higher plants, there are two distinct routes to biosynthesize isopentenyl diphosphate (IPP) and its isomer dimethylallyl diphosphate (DMAPP) which are the central five-carbon precursors of all isoprenoids: the MVA pathway in the cytosol and the 2-methylderythritol-4-phosphate (MEP) pathway in plastids^29–31^. The MVA pathway predominantly synthesizes sesquiterpenoids, triterpenoids such as sterol, ubiquinones, and other polyterpenoids^32,33^. Separately, the MEP pathway mainly involves the biosynthesis of monoterpenoids, diterpenoids and other terpenoids such as hormones, plant pigments and plastoquinone^34^. Since santalols are sesquiterpenoids, we focused on the genes in the MVA metabolic pathway. Mevalonate kinase (MK, ATP: mevalonate-5-phosphotransferase; E.C. 2.7.1.36), which is the fourth enzyme in the MVA metabolic pathway, catalyzes the conversion of MVA into mevalonate-5-phosphate. Phosphomevalonate kinase (PMK, E.C. 2.7.4.2) then catalyzes the conversion of mevalonate-5-phosphate into mevalonate-5-diphosphate. MK belongs to the GHMP superfamily and catalyzes the first phosphorylation reaction in the MVA pathway^35^. Studies have shown that geranyl diphosphate (GPP), farnesyl diphosphate (FPP) and geranylgeranyl diphosphate (GGPP) can inhibit MK activity^36^. Since FPP, GPP and GGPP are important precursors of terpenoid synthesis, MK can play an important role in regulating the biosynthesis of terpenoids^37,38^. PMK, which also belongs to the GHMP superfamily^35^ and is a potential regulatory enzyme of the isoprenoid biosynthetic pathway, can be expressed at relatively low levels and may be a target for increasing overall isoprenoid production^39–41^.

Recently, some *MK* genes have been isolated from various plant species, such as *Hevea brasiliensis*^42^, *Agave americana*^43^, *Arabidopsis thaliana*^36^, *Catharanthus roseus*^44^, *Zea mays*^45^ and *Ginkgo biloba*^46^. Some *PMK* genes have been isolated from various plant species, such as *A. thaliana*^47^, *H. brasiliensis*^48^ and *Salvia miltiorrhiza*^49^. However, little is known about the genes encoding *MK* and *PMK* in *S. album* or the mechanism regulating their expression. Overexpression of *MK* and *PMK* genes in *Escherichia coli* DH10B significantly increased MK and PMK protein levels, as well as an over three-fold increase in amorpha-4,11-diene^50^. Therefore, an analysis of *MK* and *PMK* genes and their functions is important to be able to further study santalol biosynthesis in *S. album*.

In the present study, two novel *MK* and *PMK* cDNAs, named as *SaMK* and *SaPMK*, respectively, were cloned and characterized from *S. album* by rapid amplification of cDNA ends (RACE) technology for the first time. Their structure and function were assessed by a bioinformatics analysis and yeast complementation assays. In addition, the expression profiles of *SaMK* and *SaPMK* were examined in various tissues (roots, shoots, young leaves, mature leaves, sapwood and heartwood). The expression patterns of *SaMK* and *SaPMK* following the induction by MeJA were also investigated.

## Results

### Cloning and characterization of the full-length cDNA of *SaMK* and *SaPMK*

The full-length cDNA sequences of *SaMK* and *SaPMK* were obtained through RT-PCR and 5′/3′ RACE. The full-length of *SaMK* is 1409 bp and contains a 1170 bp open reading frame (ORF) that encodes 389 deduced amino acid residues. The full-length of *SaPMK* is 1679 bp and contains a 1527 bp ORF that encodes 508 deduced amino acid residues. The results of BLASTN analysis on NCBI revealed that the *SaMK* and *SaPMK* sequences were highly homologous to *MK* and *PMK* genes from other plants (Table 1). The *SaMK* nucleotide sequence exhibited 76%, 75%, 75%, 74% and 73% similarity with *H. brasiliensis*, *Morus alba*, *Platycodon grandiflorus*, *Panax notoginseng* and *C. roseus*, respectively. The *SaPMK* nucleotide sequence exhibited 76% similarity with *Tripterygium wilfordii*, *M. alba* and *H. brasiliensis* and 74% similarity with *P. ginseng* and *Siraitia grosvenorii*. Therefore, these genes were designated as *SaMK* (GenBank accession No. MH018696) and *SaPMK* (GenBank accession No. MH018697).

**Table 1:** Nucleotide sequences of *SaMK* and *SaPMK* and similarity to genes from other plant species.

Species	Accession number	Identity (%)
***MK***		
*Hevea brasiliensis*	JN036543.1	76
*Morus alba*	KX387386.1	75
*Platycodon grandiflorus*	KC439364.1	75
*Panax ginseng*	JQ957844.1	74
*Catharanthus roseus*	HM462019.1	73
***PMK***		
*Tripterygium wilfordii*	KR260990.1	76
*Morus alba*	KX387387.1	76
*Hevea brasiliensis*	JN036535.1	76
*Panax ginseng*	KJ804170.1	74
*Siraitia grosvenorii*	HQ128558.1	74

### Bioinformatics analysis of the deduced SaMK and SaPMK proteins

**Figure 1:**
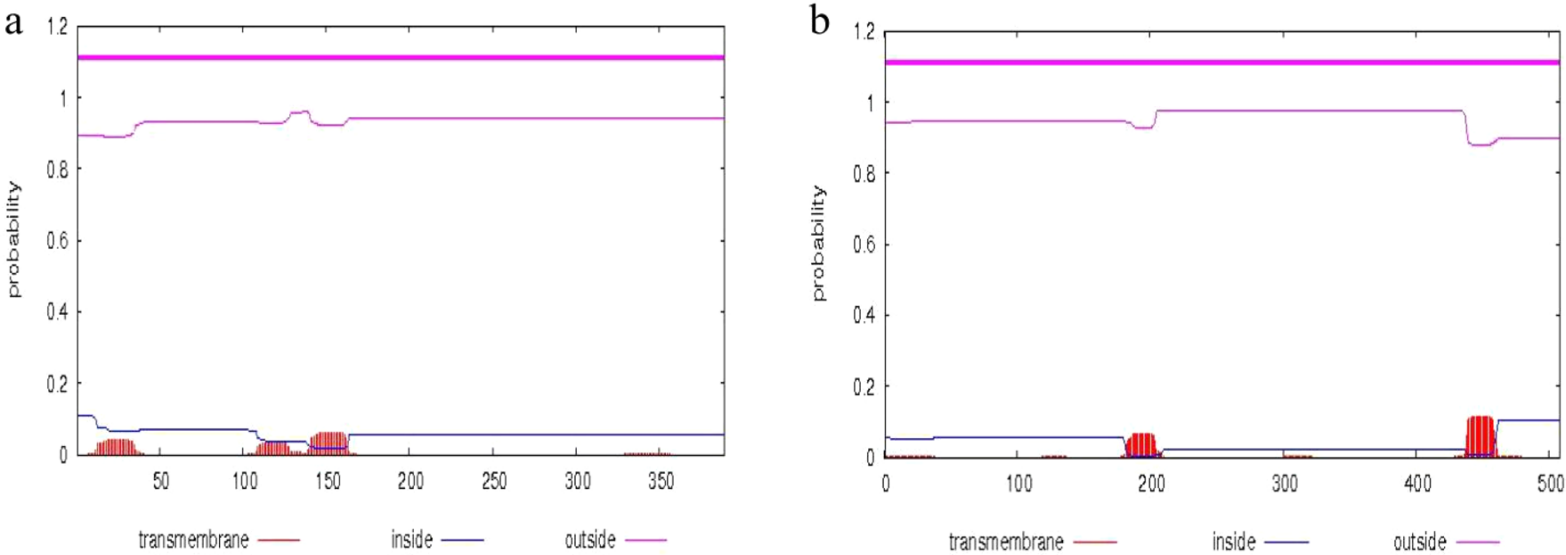
Prediction of transmembrane domains of SaMK (**a**) and SaPMK (**b**) proteins in *Santalum album*.

The ExPASy online tool was used to calculate the physicochemical properties of the deduced SaMK and SaPMK proteins. The results are shown in Table 2. The predicted relative molecular weight (MW) of the SaMK protein is 41.3 kDa and the relative MW of the SaPMK protein is 54.6 kDa. The theoretical isoelectric points of SaMK and SaPMK are 5.23 and 5.92, respectively. The instability index of SaMK is 33 and that of SaPMK is 46, indicating that they are both stable proteins (Table 2). The total average hydropathicity of SaMK is 0.113, indicating that it is a hydrophobic protein, while that of SaPMK is − 0.073, indicating that it is a hydrophilic protein (Table 2). Transmembrane domain and signal peptides were predicted by the TMHMM Server and SignalP, respectively. SaMK and SaPMK have no transmembrane domain or signal peptide (Fig. 1), indicating that they are non-secretory proteins.

**Table 2:** Physicochemical properties of deduced proteins in *Santalum album*.

Proteins	Molecular weight (kD)	Theoretical isoelectric point	Number of acidic amino acids	Number of basic amino acids	Instability index	Aliphatic index	Total average hydropathicity
SaMK	41.3012	5.23	42	33	32.42	99.02	0.113
SaPMK	54.5954	5.92	52	46	32.87	89.84	− 0.073

### Predicted protein structure and functional domain

**Figure 2:**
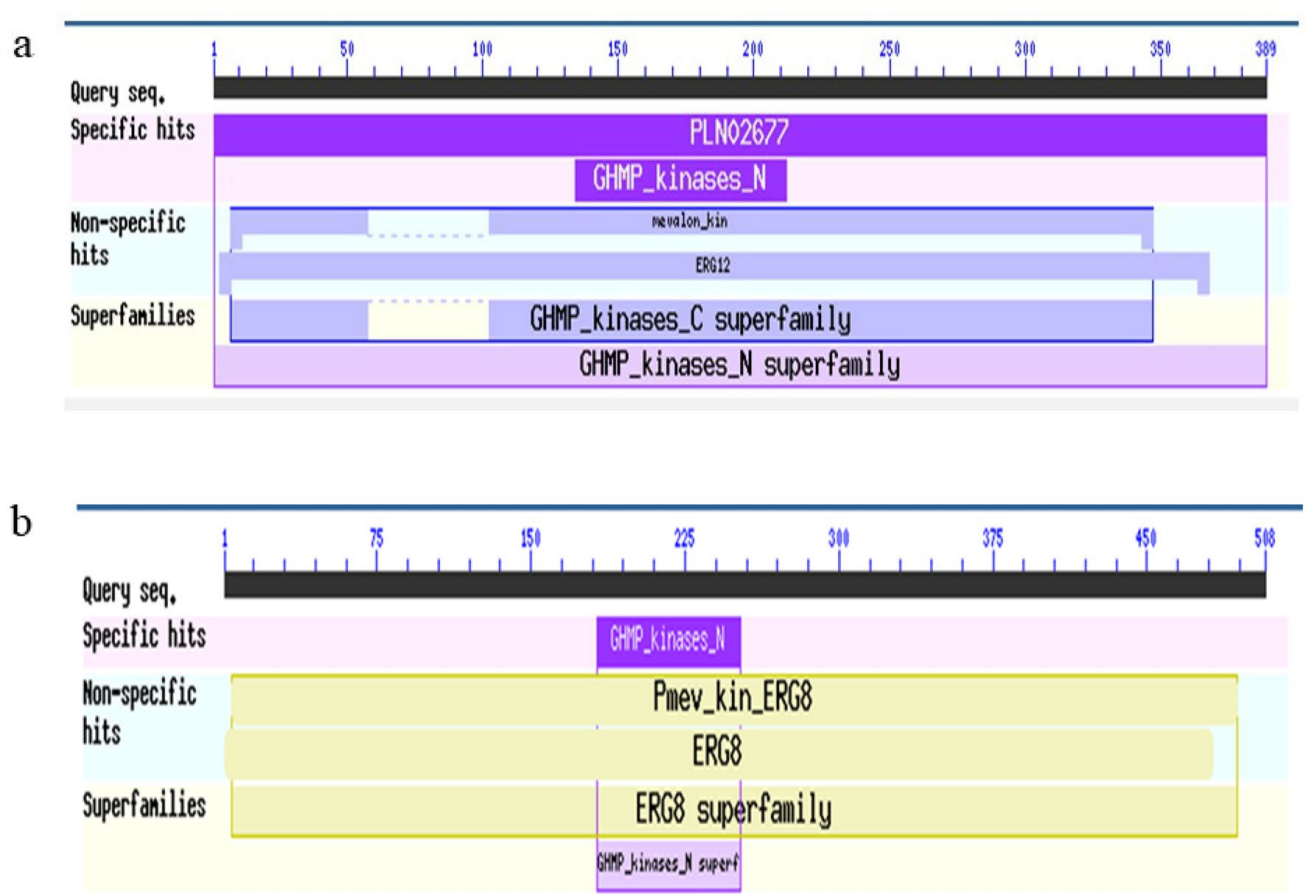
Conserved domains of SaMK (**a**) and SaPMK (**b**) proteins in *Santalum album*.

**Figure 3:**
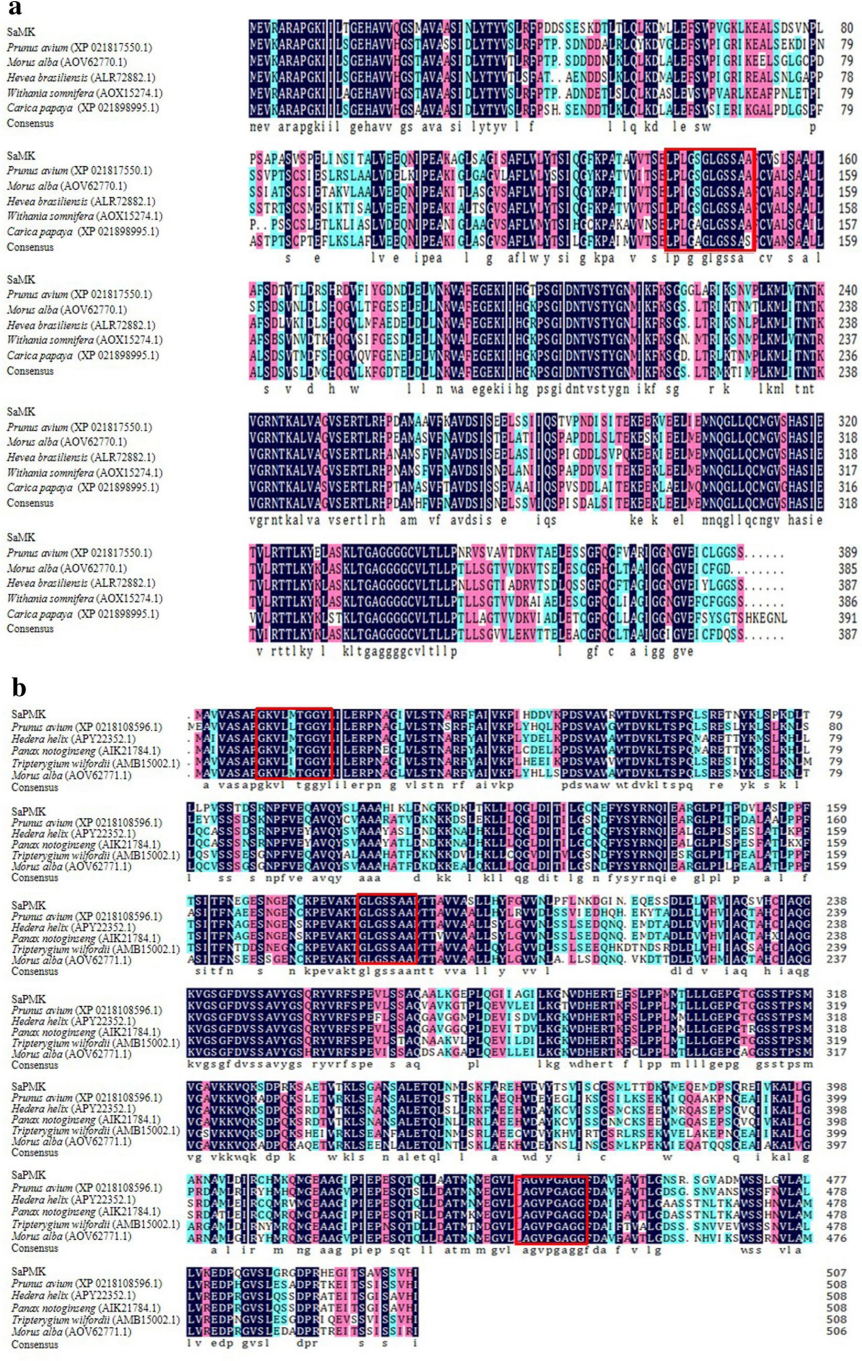
Multiple alignments of SaMK (**a**) and SaPMK (**b**) deduced amino acid sequences in *Santalum album* with other corresponding homologous proteins. Red frames indicate conserved motifs.

The amino acid sequences deduced from *SaMK* and *SaPMK* genes were analyzed by the NCBI Conserved Domains database (Fig. 2). The SaMK protein contains the N-terminal conserved region of GHMP kinase (GHMP) from amino acids 134–212 and the C-terminal conservative region from amino acids 296–365 (Fig. 2a). SaMK has significant MK activity from amino acids 1–389 and SaPMK has significant PMK activity from amino acids 1–482 (Fig. 2b). The active site of MK exists between amino acids 139 and 150 (LPLGSGLGSSAA) in *SaMK* and is an ATP binding domain sequence of GHMP kinase (Fig. 3a). SaPMK (Fig. 3b) also contains the N-terminal conserved region of GHMP kinase from amino acids 182–252 and has three conserved motifs, GKVLLAGGY (10–18), GLGSSA (187–193) and GGGVPGAGG (448–456). These findings confirmed that SaMK and SaPMK have similar catalytic functional domains to the corresponding MK and PMK from other species.

### Molecular evolution of the deduced SaMK and SaPMK proteins

**Figure 4:**
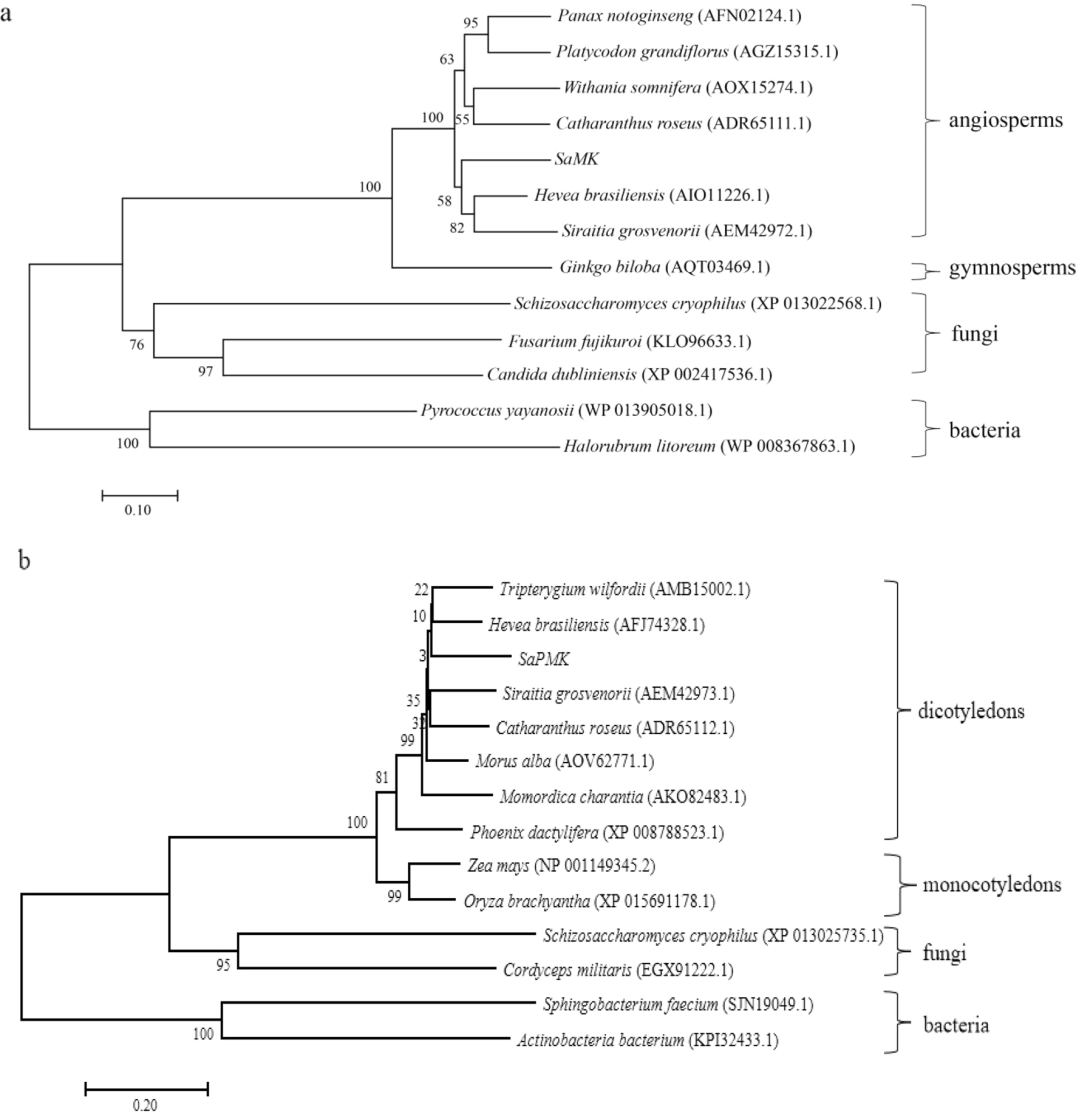
Phylogenetic trees of SaMK and SaPMK proteins in *Santalum album* relative to other organisms. (**a**) SaMK; (**b**) SaPMK.

To investigate the evolutionary relationships among deduced SaMK and SaPMK proteins with other MKs and PMKs from angiosperms, gymnosperms, fungi, and bacteria, phylogenetic trees were constructed using the NJ method with MEGA 7. As shown in Fig. 4a, MKs from different species appeared to evolve into four groups, with bacteria as an ancient group. SaMK belonged to the angiosperms group and was clustered into one group with *Siraitia grosvenorii* and *H. brasiliensis.* As shown in Fig. 4b, PMKs from different species also evolved into four groups with bacteria as the ancient group. SaPMK was in the same group with dicotyledons and was clustered into one group with *H. brasiliensis* and *Tripterygium wilfordii*. These results suggest that SaMK and SaPMK shared a common evolutionary origin with MK and PMK proteins of other plants.

### Subcellular localization of SaMK and SaPMK proteins

**Figure 5:**
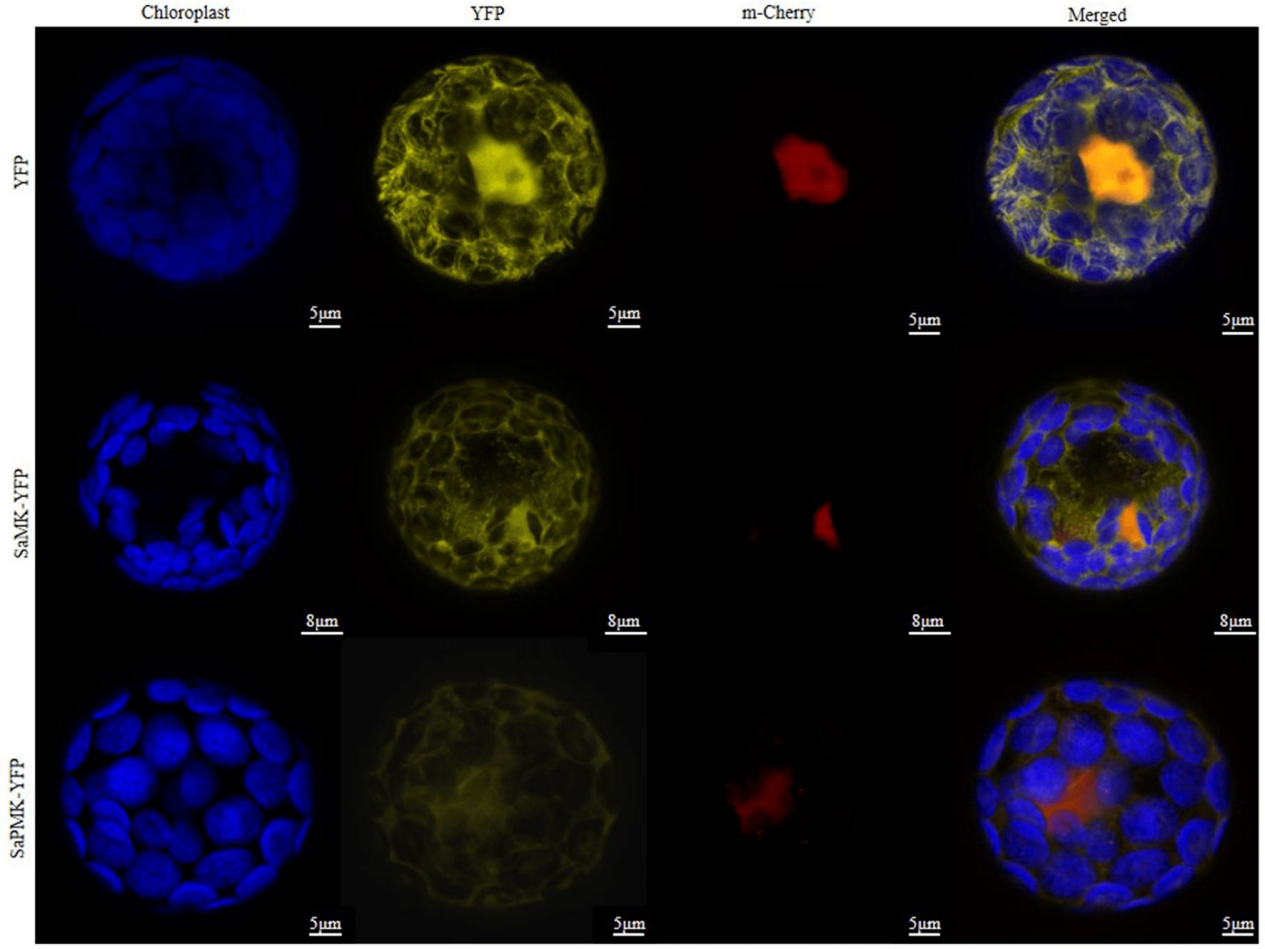
Subcellular localization of *Santalum album* SaMK and SaPMK. Blue fluorescence indicates chlorophyll (Chl) autofluorescence, yellow fluorescence indicates YFP and fusion vectors signal, and red signal indicates m-Cherry fluorescence. The merged images represent a digital combination of Chl autofluorescence, YFP fluorescent and m-Cherry protein fluorescence. Scale bars: SaPMK-YFP and YFP = 5 μm; SaMK-YFP = 8 μm.

To further verify the subcellular localization of SaMK and SaPMK, subcellular localization of SaMK-YFP and SaPMK-YFP (yellow fluorescent protein) were studied using a modified polyethylene glycol method to transform SaMK-YFP and SaPMK-YFP constructs into *A. thaliana* protoplasts. It was found that both SaMK and SaPMK were located in the cytosol (Fig. 5).

### Functional complementation of SaMK and SaPMK in *Saccharomyces cerevisiae*

**Figure 6:**
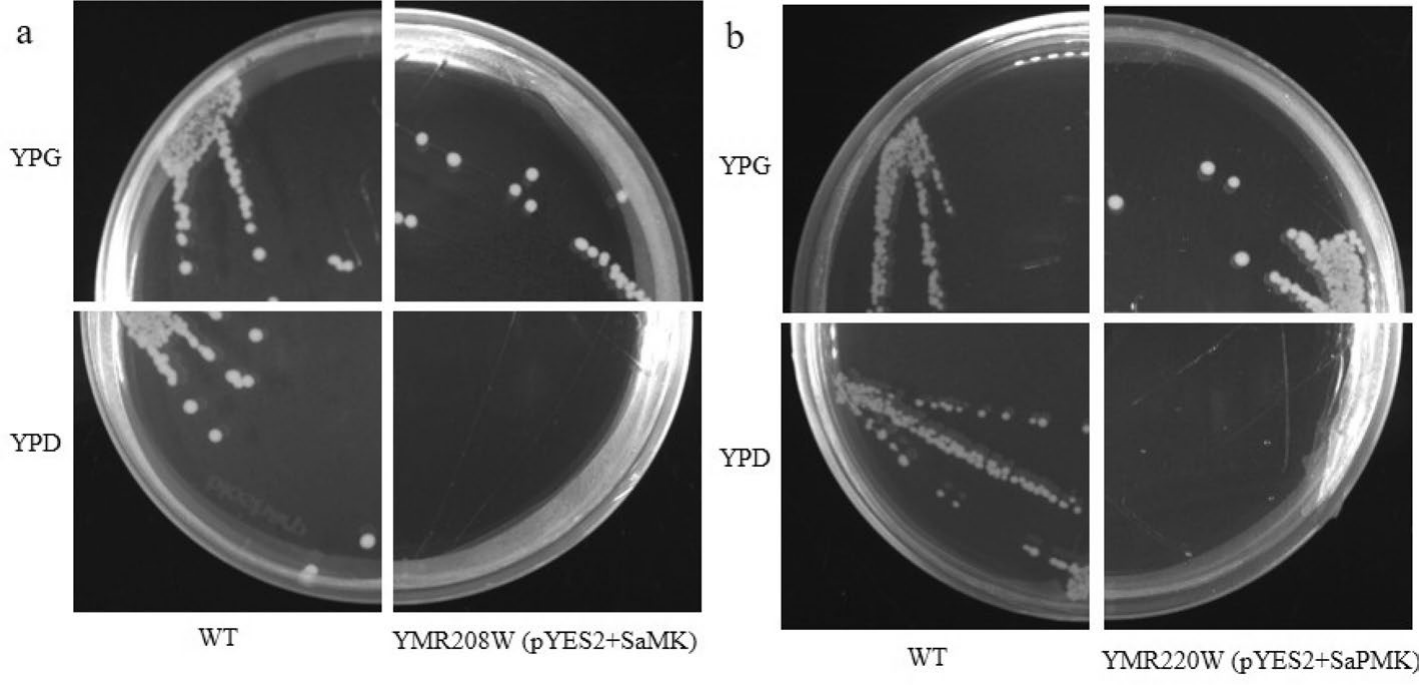
Functional complementation of *SaMK* (**a**) and *SaPMK* (**b**) genes in *Santalum album*. The strains were grown on YPG and YPD medium at 30 °C for 3 days with the exception of YMR208W with *SaMK* and YMR220W with *SaPMK*.

Disrupting MVA pathway genes in yeast strains can be fatal^51,52^. To verify the function of *SaMK* and *SaPMK*, two recombined expression vectors, pYES2-SaMK and pYES2-SaPMK, were successfully constructed. YMR208W, which harbored pYES2-SaMK, and YMR220W, which harbored pYES2-SaPMK, grew well on YPG medium. However, neither YMR208W, which harbored pYES2-SaMK, nor YMR220W, which harbored pYES2-SaPMK, could grow on YPD medium (Fig. 6). These results indicate that SaMK and SaPMK have MK and PMK activity, respectively.

### Tissue-specific expression of *SaMK* and *SaPMK*

**Figure 7:**
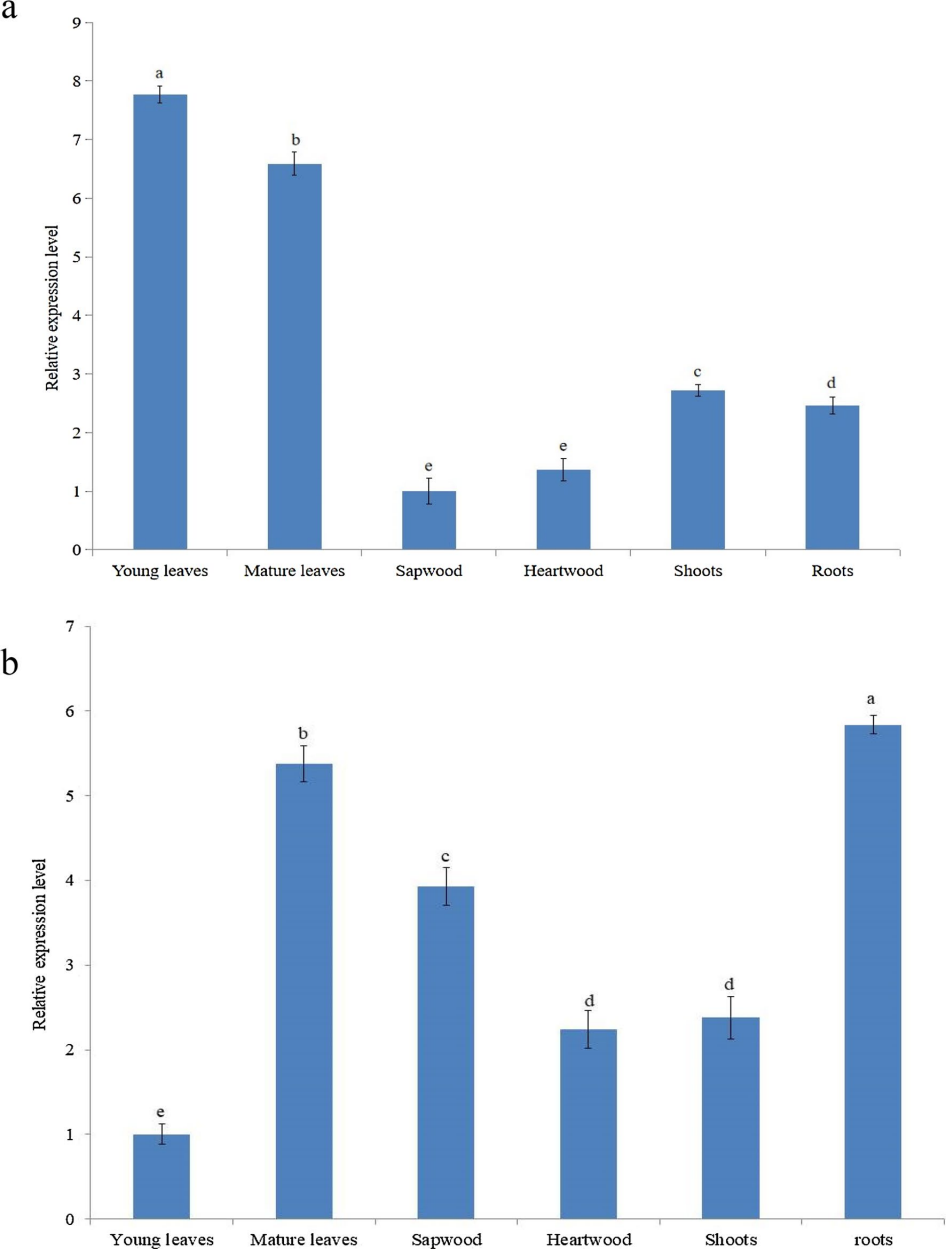
Tissue expression analysis of *SaMK* (**a**) and *SaPMK* (**b**) genes in *Santalum album*. The gene expression level of *SaMK* in sapwood and *SaPMK* in young leaves were set to 1. Data from qRT-PCR are means ± SD (standard deviation) from triplicate experiments (n = 3). Different letters indicate significant differences (p < 0.05) according to Duncan’s multiple range test.

To determine the tissue-specific expression patterns of *SaMK* and *SaPMK* genes in *S. album*, total RNA was extracted from roots, heartwood, sapwood, young leaves, mature leaves and shoots, and qRT-PCR was performed. The results of qRT-PCR are shown in Fig. 7. *SaMK* and *SaPMK* were constitutively expressed in all tissues of *S. album*. As shown in Fig. 7a, the lowest level of *SaMK* transcript was observed in sapwood, and the highest expression level in young leaves followed by mature leaves and shoots, approximately 7.77-, 6.59- and 2.72-fold higher than in sapwood. The expression level of *SaPMK* (Fig. 7b) was lowest in young leaves but was highest in roots followed by mature leaves and sapwood, approximately 5.84-, 5.38- and 3.93-fold higher than in young leaves.

### Expression of *SaMK* and *SaPMK* in response to MeJA

**Figure 8:**
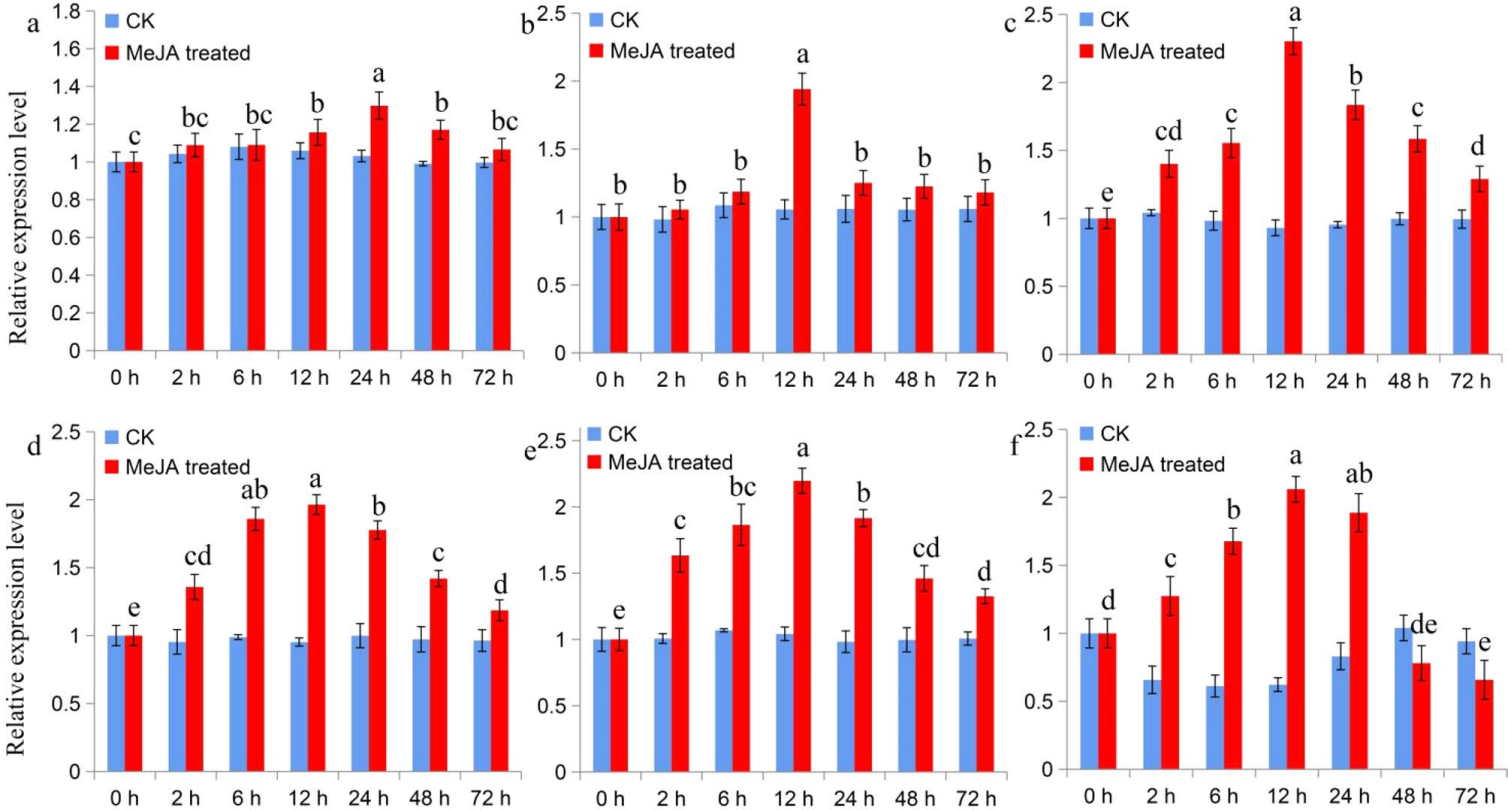
Level of *SaMK* and *SaPMK* transcripts in *Santalum album* roots (**a**: *SaMK*, **d**: *SaPMK*), shoots (**b**: *SaMK*, **e**: *SaPMK*) and leaves (**c**: *SaMK*, **f**: *SaPMK*) after induction with MeJA. The gene expression level of *SaMK* and *SaPMK* (untreated control) was set to 1. Data from qRT-PCR are means ± SD (standard deviation) from triplicate experiments (n = 3) and different letters indicate significant differences (p < 0.05) according to Duncan’s multiple range test.

MeJA is a plant-specific signaling molecule that is involved in the regulation of various biological processes^53^. In the present study, we measured the expression level of *SaMK* and *SaPMK* in *S. album* roots, shoots and leaves after treatment with 100 μM MeJA (Fig. 8). The expression of both genes was significantly induced by MeJA. The change in transcript level of *SaMK* and *SaPMK* in *S. album* roots, shoots and leaves after MeJA treatment was consistent, all increasing gradually and peaking at 12 h and then gradually decreasing compared with control seedlings. However, the level of increase in different tissues differed.

## Discussion

Terpenoids, including monoterpenes, sesquiterpenes and diterpenes, play an important role in plant physiology and ecology^54^. In recent years, many studies have documented the molecular regulation of sesquiterpene biosynthesis^55^. Santalol, a sesquiterpenoid, is the most dominant aromatic and active ingredient in sandalwood essential oil^7^. Santalol is mainly synthesized via the MVA pathway. The functions of MK and PMK proteins in the MVA pathway have been studied in many plant species^36,44^. MK expression level is related to the precursors of terpenoid biosynthesis, IPP and DMAPP, which can indirectly regulate the biosynthesis of terpenoids, and overexpression of the *PMK* gene can increase the content of terpenoids^56^. However, previous reports on sandalwood terpenoids mainly focused on downstream enzymes such as sesquiterpene synthase, which can yield sesquiterpene hydrocarbons^57–60^, and cytochrome P450 oxygenase, which converts sesquiterpene hydrocarbons to corresponding sesquiterpene alcohols^24,61^. Only two genes upstream of the MVA pathway, *AACT* and *HMGS*, have been reported^62^. Other than this study, there are currently no reports of *MK* and *PMK* genes in *S. album.* Thus, we attempted to examine the molecular biology of the MVA pathway via santalol biosynthesis in *S. album* by cloning, characterization, and functional analysis of full-length cDNAs of *SaMK* and *SaPMK* genes.

In this study, a 1409 bp full-length cDNA of the *SaMK* gene and a 1679 bp full-length cDNA of the *SaPMK* gene were isolated from mature leaves of *S. album*. The deduced SaMK protein contained 389 amino acids and weighed 41.30 kDa, which is consistent with the predicted MW of the *A. thaliana* MK protein, which is 40.7 kDa^36^. The deduced SaPMK protein contained 508 amino acids and weighed 54.60 kDa. The MW of PMKs from different species vary widely^44,51,63^. The relative MW of the SaPMK protein is similar to the PMK protein of *M. chamomilla*. Multiple alignments showed that the deduced SaMK and SaPMK protein sequences were very similar to other plant MKs and PMKs, respectively. Moreover, the conserved motifs of SaMK and SaPMK proteins were consistent with previous studies in *Enterococcus faecalis* and *Streptococcus pneumoniae*^64,65^. These findings indicate that *SaMK* and *SaPMK* have similar catalytic functions to other plant MVKs and PMKs. A phylogenetic tree revealed that the SaMK protein had highest homology with MKs from *H. brasiliensis* and *Siraitia grosvenorii* while the SaPMK protein had highest homology with PMKs from *H. brasiliensis* and *Tripterygium wilfordii*. This trend indicates that SaMK and SaPMK shared common evolutionary origins with other MK and PMK proteins based on their amino acid sequences and functional domains. In *C. roseus*, the MK protein is located in the cytosol while the PMK protein is located in peroxisomes^66^. However, predicted subcellular localization of SaMK and SaPMK by PSORT showed that they might be localized in the cytoplasm and results of a modified polyethylene glycol method verified that SaMK and SaPMK are both localized in the cytosol, suggesting that *SaMK* and *SaPMK* cloned in this study may be involved in the MVA pathway in *S. album*. *SaMK* and *SaPMK* complementation assays in yeast revealed that their expression provided basic nutrients for the survival of yeast, thereby confirming the catalytic function of SaMK and SaPMK proteins^46,51^.

Sandalwood is considered to be one of the most valuable trees in the world^67^. Its value lies mainly in its heartwood and the essential oil extracted from heartwood^3^. Santalol is responsible for the pleasant fragrance of sandalwood^13^ and most of the oil’s pharmacological activity^68^. Thus, it is important to investigate whether or not *SaMK* and *SaPMK* transcripts may be related to the accumulation of santalol in different *S. album* tissues. qRT-PCR showed that *SaMK* and *SaPMK* genes were constitutively expressed in all the tested tissues, but at different levels. The *SaMK* transcript level in young leaves was significantly higher than in other tissues, and its expression level was lowest in sapwood. A similar expression pattern was observed in *Ginkgo biloba*, in which *GbMK* genes were highly expressed in roots and leaves^46^. In *H. brasiliensis*, the *HbMK* gene was highly expressed in latex, followed by xylem and mature leaves^48^. The level of the *SaPMK* transcript in roots was significantly higher than in other tissues, and its expression level was lowest in young leaves. In *H. brasiliensis*, the *HbPMK* gene was highly expressed in xylem, followed by latex^48^. In *Panax ginseng*, the *PgPMK* gene was highly expressed in fine roots, followed by lateral roots^68^. These studies revealed that *MK* and *PMK* genes may have distinct spatial and temporal expression patterns in different plant species.

MeJA is an important plant growth regulator involved in diverse developmental processes, such as seed germination, root growth, fertility, fruit ripening, and senescence^69^. Previous studies showed that there is a relationship between MeJA and terpene metabolism^70,71^. MeJA promoted the production of monoterpenoids and sesquiterpenoids in *Ocimum basilicum*^72^, *Sarcophyton glaucum*^69^, *Salvia miltiorrhiza* and *G. biloba*^73,74^. In *S. album*, MeJA induced the expression of *SaTPS1* and *SaTPS2* in leaves^24^. Thus, studying the expression profiles of *SaMK* and *SaPMK* following treatment with MeJA is important because it may provide insight into the regulation of these genes in santalol biosynthesis. In the present study, the expression levels of *SaMK* and *SaPMK* increased significantly in *S. album* roots, shoots and leaves after treatment with 100 μM MeJA, peaking at 12 h after treatment, then gradually decreasing, indicating that these inducible genes might be involved in signal molecule-related responses to environmental stimuli. The *MK* gene transcript was induced by 1 mM MeJA in *G. biloba*^46^ and the *PMK* gene transcript was induced by 100 μM MeJA in *P. ginseng*^68^.

The characterization and expression profiles of *SaMK* and *SaPMK* genes may contribute to an understanding of the biosynthesis of sesquiterpenes in *S. album* at the molecular level and the regulatory mechanisms involved in the MVA pathway.

## Materials and methods

### Plant material

Five-year-old sandalwood trees growing in South China Botanical Garden (SCBG), Guangzhou, China, were used. Permission and guidance was obtained from SCBG and the local government for using this plant material for this study. Young (light green) and mature (dark green) leaves, heartwood, sapwood, roots and shoots were collected and wrapped in tin foil, frozen immediately in liquid nitrogen, and stored at − 80 °C for subsequent analyses. Two-month-old young seedlings (6–8 leaves) of *S. album* were sprayed with 100 μM MeJA (dissolved in 2% ethanol) until the leaf surfaces were wet, and 2% ethanol served as the control for each treatment. Samples (leaves, shoots and roots) were collected at 0, 2, 6, 12, 24, 48 and 72 h after treatment and stored at − 70 °C for further analyses. Each treatment was repeated three times.

### Cloning of the full-length putative cDNA of *SaMK* and *SaPMK* by RACE

Total RNA of mature sandalwood leaves was extracted using Column Plant RNAOUT (Tiandz, Beijing, China) according to the manufacturer’s instructions. The concentration and quality of RNA were measured using a NanoDrop ND-1000 spectrophotometer (Nanodrop Technologies, Wilmington, NC, USA) and agarose gel electrophoresis. First-strand cDNA was synthesized by the PrimeScript first-strand cDNA synthesis kit (Takara Bio Inc., Dalian, China). 5′ and 3′ RACE were performed with the SMARTer RACE cDNA amplification kit (Clontech Laboratories Inc., Mountain View, CA, USA) manual. Primers were designed on the basis of initial data of MK and PMK unigenes in the transcriptome^75^ (Table 3). The sequence information of 5′ and 3′ RACE PCR product clones were used to design primers from the start and stop codon to obtain the internal fragments. The amplified PCR products were purified by a gel DNA purification kit (Tiangen, Beijing, China) and ligated into the pMD18-T vector (Takara Bio Inc.). The recombined plasmids were transformed into *Escherichia coli* DH5α competent cells (Takara Bio Inc.) and sequenced at the Beijing Genomics Institution (BGI, Shenzhen, China).

**Table 3:** Primers used for related experiments in this study.

Primer purpose	Primer name	Primer sequence (5′ → 3′)
5′ RACE	MK-5(1)	CGCAGATGAACCCAATCCAGAAC
	MK-5(2)	CCTCGGGAATGTTCTGCTCTTCA
	PMK-5(1)	TGTCAGAGGGAGCCCACGTGCCT
	PMK-5(2)	AAGAGTACTGCACCGCTTGTTCT
3′ RACE	MK-3(1)	CGACAACACGGTCAGCACATA
	MK-3(2)	AATGAATCAGGGGTTGCTCCA
	PMK-3(1)	ATGGCTGTAGTTGCTTCTGCTCC
	PMK-3(2)	GTGAAACTAACATCTCCTCAGCTC
ORF	MK-O(F)	ATGGAGGTGAGGGCTCGAGCTC
	MK-O(R)	TGAAGAACCACCGAGACAAATCT
	PMK-O(F)	ATGGCTGTAGTTGCTTCTGCTCC
	PMK-O(R)	TCCAATGTGAACTGAAGAAACAG
qRT-PCR	q-MK-F	GCTTCCTCTAGGTTCTGGATTG
	q-MK-R	CTGCGGTCCAAAGTTACTGTAT
	q-PMK-F	GACTGGCGGTTACCTCATTT
	q-PMK-R	GCTTGACATCATCGTGAATTGG
Functional complementation	pYES2-MK-F	CCGGAATTCGAATGGAGGTGAGGGCTCGAGCTC
	pYES2-MK-R	TAAAGCGGCCGCGTGAAGAACCACCGAGACAAATCT
	pYES2-PMK-F	CCGGAATTCGAATGGCTGTAGTTGCTTCTGCTCC
	pYES2-PMK-R	TAAAGCGGCCGCGTCCAATGTGAACTGCTGAAG
Subcellular localization	YFP-MK-F	CCGGAATTCATGGAGGTGAGGGCTCGAGCTC
	YFP-MK-R	TCCCCCGGGTGAAGAACCACCGAGACAAATCT
	YFP-PMK-F	CCGGAATTCATGGCTGTAGTTGCTTCTGCTCC
	YFP-PMK-R	CGCGGATCCATCCAATGTGAACTGAAGAAACA

### Bioinformatics analysis and molecular evolution analysis of *SaMK* and *SaPMK*

*SaMK* and *SaPMK* gene sequences were assembled and translated into amino acid sequences using DNAMAN software. The ORFs of *SaMK* and *SaPMK* genes were predicted by ORFfinder (https://www.ncbi.nlm.nih.gov/orffinder/). Sequence comparison was performed with NCBI BLAST online tools (http://www.ncbi.nlm.nih.gov/BLAST/). Physicochemical properties such as MW, theoretical isoelectric point, instability index, aliphatic index and grand average of hydropathicity of the deduced SaMK and SaPMK proteins were calculated by ExPASy (http://cn.expasy.org). Protein domains and active sites were predicted by the CDD database in NCBI (http://www.ncbi.mlm.nih.gov/Structure/cdd/wrpsb.cgi). Transmembrane domains and signal peptides were predicted by the TMHMM Server (http://www.cbs.dtu.dk/services/TMHMM/) and SignalP (http://www.cbs.dtu.dk/services/SignalP/), respectively. Sub-cellular localization was predicted by the PSORT online tool (http://www.psort.org/). Multiple sequence alignment was performed with CLUSTALX 2.0 (Conway Institute, UCD Dublin, Dublin, Ireland) and phylogenetic trees of SaMK and SaPMK proteins from *S. album* and other plants were constructed by MEGA 7 using the neighbor-joining (NJ) method with 1000 bootstrap replicates^76^.

### Subcellular localization of SaMK and SaPMK proteins

A vector pSAT6-EYFP containing the enhanced yellow fluorescent protein (EYFP) ORF was used in this study. The cDNAs encoding SaMK and SaPMK were amplified with two pairs of primers, YFP-MK-F and YFP-MK-R, and YFP-PMK-F and YFP-PMK-R, respectively (Table 1). The PCR product of MK was digested with *Eco*RI and *Sam*I, the PCR product of PMK was digested with *Eco*RI and *Bam*HI, and the pSAT6-EYFP vectors were digested with corresponding restriction endonucleases. The digested fragments were ligated into the linearized pSAT6-EYFP vector to generate pSAT6-EYFP-SaMK and pSAT6-EYFP-SaPMK fusion constructs. The fusion expression vectors and the pSAT6-EYFP vector were transformed into *A. thaliana* mesophyll protoplasts through PEG-mediated transformation following a previously described method^77^. A confocal laser-scanning microscope (Leica TCS SP8 STED 3X, Wetzlar, Germany) was used to observe YFP fluorescence in transformed protoplasts after overnight incubation in a constant temperature incubator (SPH-2102C, Shanghai, China) at 22 °C. Fluorescence was excited for YFP at 514 nm, for Chl at 543 nm and for m-Cherry at 587 nm.

### Functional complementation of *SaMK* and *SaPMK* in yeast

The two recombined plasmids, pYES2-SaMK and pYES2-SaPMK, were constructed by the In-Fusion HD Cloning Kit (Takara Bio Inc.) according to the manufacturer’s instructions. The pYES2 vectors (Invitrogen, Carlsbad, CA, USA), which contained a yeast galactose-dependent promoter that can promote high levels of expression of target genes, were used as carriers for target genes in this study. The recombined plasmids (pYES2-SaMK and pYES2-SaPMK) were extracted and transformed into YMR208W (ΔERG12) and YMR220W (ΔERG8) (Dharmacon, Chicago, IL, USA), respectively with the Frozen-EZ Yeast Transformation II Kit (Zymo Research, Irvine, CA, USA). Transformants were spotted on SC (-Ura) medium (6.7% yeast nitrogen base without amino acids, 2% galactose)^46^. Positive clones were further confirmed by PCR. Subsequently, transformed diploid cells were induced to sporulate and formed haploid cells containing pYES2-SaMK and pYES2-SaPMK. To further observe their growth, the diploid *Saccharomyces cerevisiae* strain YSC1021 and transformed haploid strains YMR208W and YMR220W were grown separately on YPD (1% yeast extract, 2% bacto peptone, 2% glucose) and YPG (1% yeast extract, 2% bacto peptone, 2% galactose) media, respectively^78,79^.

### Tissue-specific analysis and expression profiles of *SaMK* and *SaPMK* induced by MeJA

To investigate the expression levels of *SaMK* and *SaPMK* genes in different tissues (roots, sapwood, heartwood, young leaves, mature leaves and shoots) and their expression profiles after MeJA treatment, qRT-PCR was carried out according to the manufacturer’s instructions. About 1.0 μg of total RNA was reverse transcribed into first-strand cDNA using the PrimeScript RT reagent kit (Takara Bio Inc.) according to the manufacturer’s protocols. The reactions were performed by ABI7500 fluorescence quantitative PCR (Applied Biosystems, Thermo Fisher Scientific, Waltham, MA, USA) using iTaq Universal SYBR Green supermix as the buffer (Applied Biosystems). The housekeeping gene, β-actin, was selected as the internal control^75^ for the normalization of all reactions. All experiments were performed in triplicate and mean values were analyzed. Significant differences (p < 0.05) between means were tested with Duncan’s multiple range test. The 2^−ΔΔCT^ method was used to analyze the relative expression level of genes^80^.

## Data Availability

All data generated or analyzed during this study are included in this published article.
